# Integration of Cardiovascular Disease Services within Primary Health Care Systems: Insights from South Asia

**DOI:** 10.1007/s11883-026-01443-x

**Published:** 2026-09-17

**Authors:** Joseph H Collins, Syed Masud Ahmed, Kaushik Chattopadhyay, Rakesh Parashar, Dinesh Neupane, Susie Perera, Zafar Mirza, Victory Olaniyi, Hasan Zaidi, Usha Konsani, Shehla Zaidi

**Affiliations:** 1 Global Business School for Health, University College London, London, UK; 2 James P Grant School of Public Health, Brac University, Dhaka, Bangladesh; 3 Lifespan and Population Health, School of Medicine, University of Nottingham, Nottingham, UK; 4 Nottingham JBI Centre for Evidence-Based Healthcare, Nottingham, UK; 5 Independent Consultant, Delhi, India; 6 Department of International Health, Johns Hopkins University, Baltimore, USA; 7 Former Deputy Director General, Independent Researcher, Ministry of Health Sri Lanka, Colombo, Sri Lanka; 8 Shifa Tameer-e-Millat University, Islamabad, Pakistan; 9 School of Medicine, Imperial College London, London, UK

**Keywords:** Primary Health Care, Cardiovascular Disease, Integrated Care, South Asia

## Abstract

**Purpose of review:**

This review assesses progress towards integrating cardiovascular disease (CVD) services within primary health care (PHC) systems in South Asia. The review examines the policy landscape, existing implementation efforts, barriers and enablers to integrated care, and the impacts of these approaches on health and health service outcomes.

**Recent findings:**

National policies widely prioritise integration in response to rising non-communicable disease burdens, but implementation remains uneven across countries. Our findings illustrate that both national programmes and smaller pilot initiatives demonstrate potential to improve screening, follow-up, treatment adherence, and address intermediate CVD risk factors such as blood pressure control. However, deficient referral systems and fragmented PHC delivery with limited engagement with the private sector continues to constrain progress. Evidence evaluating impacts of integrated care on CVD incidence and mortality remains limited.

**Summary:**

Integrating CVD services within PHC is a clear policy priority in the region, yet translating political commitment into effective implementation remains challenging requiring novel approaches integrating service provider networks, embedding digital and home support innovations and referral pathways. Future research should prioritise robust evaluation of the impacts on CVD morbidity and mortality.

## Introduction

South Asia, home to a quarter of the world’s population, is experiencing a profound health transition. Significant economic growth [1, 2], and national policy attention have reduced maternal and child mortality and communicable diseases [3] resulting in an extended life expectancy. This shift, compounded by rapid urbanisation [4], has increased vulnerability to non-communicable diseases (NCDs) [3], with cardiovascular disease (CVD) now the leading cause of premature death due to a complex interplay of social determinants and genetic predisposition [5] (Box 1). Economic development and globalisation have reshaped dietary patterns, with increased consumption of energy-dense foods high in saturated fat, sugar, and salt [6]. At the same time, the depletion of green spaces in rapidly expanding urban centres has contributed to increasingly sedentary lifestyles [7]. Patterns of rural-to-urban migration further amplify these risks, as low-paid migrants often exchange previously healthier lifestyles for urban environments characterised by workplace hazards, precarious employment, overcrowded living conditions [8], and greater exposure to high levels of pollution [9].

Box 1 – Overview of CVD epidemiology in South Asia.

**Table:** 

• Age-standardised prevalence of CVD in South Asia is 5974 per 100,000 individuals [10]. • Metabolic risk factors for CVD (e.g. diabetes, hypertension, obesity) are present in nearly a third of the population and behavioural risk factors (e.g. smoking status, alcohol intake, low physical activity) in a quarter [11]. • Co-existence of multiple risk factors is common, and particularly high for behavioural factors (49% (95% CI: 42%–56%)) [11]. • As many as two-thirds of incident CVD cases or deaths (64% and 69% respectively) are attributable to behavioural, metabolic or environmental factors [12]. • Exposure to outdoor and indoor pollution drives regional CVD mortality with 37 of the world’s 40 most polluted cities being situated South Asia [9]. • CVD is now the leading cause of death and Disability Adjusted Life Years (DALYs) in South Asia [3] with morbidity and mortality associated with CVD is three times higher than in high-income settings [10]. • Over a fifth of deaths and DALYs attributable to CVD in 2023 occurred in South Asia [3]. • By 2050 crude cardiovascular mortality on the Asian continent is expected to nearly double (91.2% increase), despite a falling age-standardise cardiovascular mortality rate, attributable to both a growing an ageing population in the coming years [13].		

Beyond health, the social and economic consequences of CVD on South Asian populations are considerable, compounded by resource constraints [14] and weak primary health care (PHC) systems [15–17]. Poorly managed and untreated CVD is associated with substantial out-of-pocket (OOP) expenditure, increasing over time [18]. For example, evidence from India suggests that as many as half of patients receiving treatment for CVD at primary or secondary health facilities will experience catastrophic health expenditure, defined as OOP payments exceeding 10% of total consumption expenditure, while costs associated with secondary or tertiary care pushed nearly a fifth of patients below the poverty line [19].

Health systems in the region are increasingly overburdened. A key driver is the growing number of patients presenting with late-stage complications of prevalent NCDs, reflecting limited early detection and prevention, suboptimal clinical management, and weak referral pathways for timely diagnosis and treatment. Public PHC systems have traditionally been oriented towards acute, episodic care rather than the long-term coordination, treatment, and follow-up required to manage chronic conditions [20]. In addition, the private health sector, which is comprised of general practice clinics, diagnostic centres, and small hospitals, delivers the majority of curative ambulatory care [21, 22], yet remains largely overlooked in national disease control initiatives. Non-profit PHC networks are limited in most countries in the region (except for Bangladesh) and have a relatively narrow scope for the management of NCDs. Addressing CVD burden will therefore require substantial restructuring of PHC systems, alongside the allocation of adequate resources and strengthened stewardship [20, 23].

Global policy positions PHC as the “delivery home” for essential NCD care as outlined in the WHO’s Global Action plan for NCDs (2023–2030) [24]. PHC as a whole-of-society approach lends itself well to CVD care as it seeks to maximise health and well-being along the care continuum with services delivered close to communities [25]. Preceding work within WHO South-East Asia and the Office for the Eastern Mediterranean regions has helped to develop regional action plans for the prevention and control of NCDs whilst the WHO’s South-East Asia-Hearts (SEAHARTS) programme provides a technical package to improve cardiovascular health with a focus on population prevention and PHC-based treatment systems [26]. While historically, disease stewardship has been vertically organised, effective CVD control will depend on more horizontal approaches to ensure continuity and coordination of care.

As such, this review seeks to explore progress towards the integration of CVD services within PHC systems in South Asia focusing on the five most populous countries in the region: Bangladesh, India, Nepal, Pakistan, and Sri Lanka. It is framed by the following research questions:

(i) What policy measures have been developed to integrate CVD services within PHC and what drivers underpin these policies?
(ii) Which initiatives have been implemented to integrate CVD services within PHC and what are their characteristics?
(iii) What are the critical barriers to and enablers of integrating CVD services within PHC?
(iv) What are the impacts of integrated CVD services on health and health system outcomes?

**Fig. 1:**
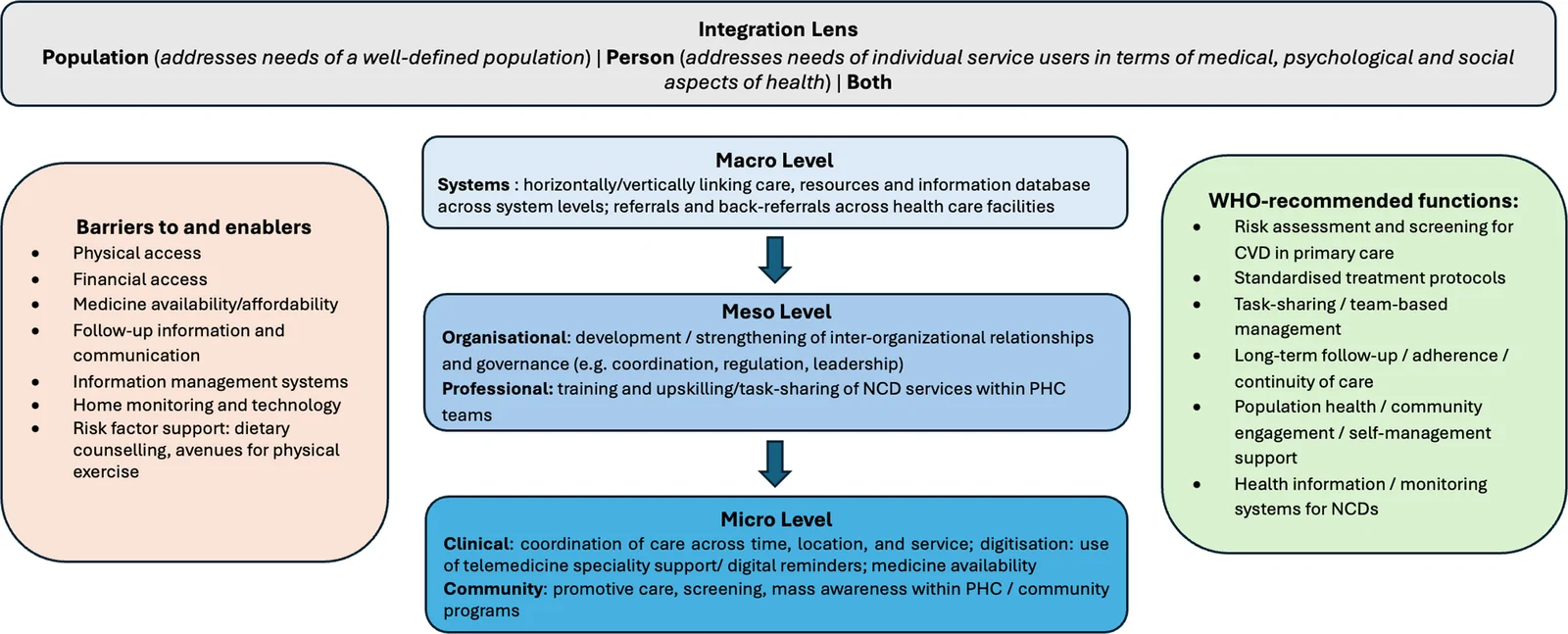
– Conceptual framework for assessing models of integration of CVD services within PHC systems

The review pulls on key programmatic-policy documentation identified by country and health system experts (SMA, KC, ZM, DN, RP, SP) to inform reflective policy insights on recent national policy initiatives, supported by a rapid scoping search of published literature (Box 2). Synthesis of findings is guided by a framework developed for this review which unpacks the integration of CVD services in PHC based on existing taxonomies of integrated PHC [27, 28] and the WHO CVD guidelines [26, 29] (Fig. 1).

Box 2 – Approach to the review of published literature.

**Table:** 

A search was conducted using the Scopus database to identify studies conducted in the last 10 years assessing the implementation of integrated approaches to CVD prevention and management within PHC. Scopus was selected due to its broad coverage and inclusion of journals indexed across health, social science, and policy disciplines. Search terms included (“PHC” OR “primary health care” Or “primary care”) AND (“cardiovascular disease” OR “CVD” OR “heart disease” or “hyperten*” OR “diabet*”) AND (“Bangladesh*” OR “India*” OR “Nepal*” OR “Pakistan*” OR “Sril Lanka*” OR “South Asia*”). Given the breadth of related review questions, a wide range of qualitative and quantitative methodological approaches were eligible for inclusion. Missing and additional relevant studies were identified by review authors.		

### What policy measures have been developed to integrate CVD services within PHC and what drivers underpin these policies?

All five South Asian countries have responded to national epidemiological transitions by demonstrating policy shifts since 2010 that seek to integrate CVD services into PHC. In some cases—notably India and Sri Lanka—this has involved broader PHC restructuring to address the rising NCD burden. Across the region, reforms have been driven by similar pressures: growing concern over CVD prevalence, fiscal constraints linked to the cost of treatment within national health insurance schemes, and commitments to universal health coverage (UHC) for expanding access to services. Policy measures have been supported politically and technically by the WHO which has led global NCD policy advocacy. In the following sections we explore the policy landscape and drivers of policy shifts across countries. Please see Table 1 for further details.

**Table 1:** Overview of the policy ecosystem relating to the integration of CVD services into PHC in South Asia

Country	Policy drivers	Policy shifts	Emphasis	Leadership of CVD-PHC	Multi-sector engagement
Bangladesh	Global advocacy, availability of local evidence, rising national concern on mortality / disability from NCDs	• Strategic Plan for Surveillance and Prevention of NCDs (2011–2015) • Multisectoral Action Plan for Prevention and Control of NCDs (2018–2025)	1. Uniform protocols 2. PEN, HEARTS pilots 3. CVD sub-package in ESP – uncertain implementation 4. Public sector PHC re-equipping	Central planning and funding by special national programme (NCD Control Programme)	Advocacy/ technical advisory support by various health stakeholders Weak institutional mechanisms for cross-sector engagement
India	National concerns to counter the epidemiological transition, escalating OOP / fiscal costs national health insurance; UHC commitments for expanded service access	• National Health Policy (2017) • India Hypertension Control Initiative (2017) • CVD integration into Ayushman Bharat PHC reform (2018) • National Multisectoral Action Plan for Prevention & Control of Common NCDs (2017–2022)	1. Protocols 2. Opportunistic population risk screening 3. NCD packages + PHC re-equipping– under expansion 4. PHC re-equipping + population empanelment for continuity of care (Ayushman Arogya Mandirs) - under expansion 5. Digitised referral data, telemedicine pilots 6. Private sector: regulation guidelines	National stewardship and small-scale HR resourcing support by National Programme for NCD Implementation and major HR resourcing by state health departments / district NCD cells Leveraging on PMJAY PHC reforms for screening, risk counselling, referrals Digital patient registry and triage by ABM initiative	Collaborations between various health stakeholders for IHCI establishment, pilots and policy best buys CVD integration into urban health policies and risk factor modification plan, absence of multisector platform, uncertain monitoring
Nepal	Increasing premature mortality and avoidable disability due to CVDs. Social economic impact of CVDs	• Multisectoral NCD Action Plan (2014–2020) • NCD and Injuries Poverty Commission (2016) • Nepal Integrated NCD Care Model (2016) • National Health Policy 2019 emphasising CVD services in PHC • Hypertension Care Cascade Program (2023) • Revamped National Multi-Sectoral Action Plan (MSAP) for NCDs 2021–2025	1. National protocols 2. PEN roll-out 3. Integration of CHWs-physicians for continuity of care 4. Digital algorithmic tools / electronic health records; individual-level risk counselling 5. Annual nationwide NCD screening campaign	Government are the leading stakeholders for CVD prevention and control with growing support from academia and NGOs	NCD commission comprising experts, government, civil society provided thought leadership, recent status unknown
Pakistan	Growing CVD burden, early onset, premature mortality. Increasing expenditure for CVD treatment. Global advocacy and UHC momentum	• National Action Plan for Prevention and Control of NCDs (2003) • National CVD Control and Health Promotion Plan (2010–2020) • National Framework for NCD Integration in PHC (2015–2020) • National Health Vision (2016-25) • National Action Framework for NCDs and Mental Health (2021-30) • National Health and Population Policy (2026-35) - Ready to be announced	1. National protocols 2. STEP survey 3. Hypertension control pilots 4. CVD interventions within EHSP – small scale implementation 5. Initial efforts for population screening – one province	National NCD unit and NCD focal persons appointed in all four provinces Integrated NCD programme in one province (Punjab) Horizontal integration of CVDs- within state/ district health	National Commission for Prevention of NCDs National task force – largely non-functional NCDs and mental health in Punjab province National Taskforce of Lifestyle Medicine - in development
Sri Lanka	Increasing burden with premature morbidity and mortality; Primary curative care system not geared to manage chronic care needs, despite distribution of primary care facilities across country; PHC reforms need a national performance framework moving on from project mode monitoring	• National Policy and Strategic Framework for Prevention and Control of NCDs (2010) • UHC policy: CVD Shared Care Cluster Model (2018) • CVD within essential health package (2019) • National guideline on dyslipidaemia management for PHC (2021) • Health policy (under development) 2026–2035 – (National Health & Wellbeing policy)	1. National CVD protocols 2. Lifestyle modification tools 3. Population based universal screening > 35 years; under 35 years with risk factors 4. Personal health record database 5. PHC re-equipping 6. Shared cluster model 7. Population empanelment to Primary care facilities	NCD Directorate in the health ministry provides technical leadership Horizontal sub-national resourcing for service delivery reorganisation. PHC being a devolved function, actual service delivery is led by decentralized units – Provincial and Regional Health Administration	Multisector engagement has been relatively weak Relevant sectors sit as official members of the NCD National steering committee chaired by health secretary National Health Development committee engages provincial stakeholders Professional bodies that also work with multi sectors and give their recommendations to the Ministry of health.

#### Bangladesh

In response to global NCD policy momentum, Bangladesh adopted the Strategic Plan for Surveillance and Prevention of NCDs (2011–2015) [30]. Under this plan, the WHO Package of Essential NCD Interventions (PEN) was piloted and a WHO STEPwise approach to NCD risk factor surveillance (STEPS) survey conducted; however, most other activities within the Strategic Plan were not implemented. A subsequent NCD Control Programme was established within the Ministry of Health and Family Welfare, introducing a PHC-focused protocol for CVD risk reduction focused on the integrated management of hypertension, diabetes, and hypercholesterolaemia [31]. Strengthened by local epidemiological evidence and growing national concern, a high-level Multisectoral Action Plan for Prevention and Control of NCDs (2018–2025) was developed and endorsed by the government [32, 33]. The plan addressed four major CVD risk factors and called for stronger inter-ministerial coordination, greater emphasis on public-sector delivery through re-equipping of PHC facilities, and exploration of financing options for an NCD sub-package within the Essential Service Package (ESP). Although the NCD Action Plan highlighted the need to improve regulation of private PHC providers, it did not articulate clear mechanisms for private-sector engagement. A multi-stakeholder CVD prevention platform, which brought together government, WHO, the National Heart Foundation, and civil society, has supported coordinated public awareness campaigns, community screening, national surveys, and evidence-informed policy development [33]. CVD implementation remains centrally funded through the NCD Control Programme, whereas the Health, Population and Nutrition Sector Program leads the ESP and broader UHC reforms. The transition towards horizontal integration therefore remains an unresolved challenge.

#### India

Domestic policy concerns driven by rapid epidemiological transition, rising costs of the national health insurance scheme, and UHC commitments to expand service access have been powerful drivers of integrated CVD services. The National Health Policy (2017) marked a conceptual inflection point, recognising CVD as a major threat to India’s health and economic development [34]. It positioned PHC as the central platform for CVD control and aligned action with the Sustainable Development Goals. The National Programme for Prevention and Control of NCDs became the principal operational vehicle for integration [35], introducing the India Hypertension Control Initiative (IHCI) in 2017 alongside NCD service packages and population-based risk screening [36, 37]. The most significant structural reform came with Ayushman Bharat–PMJAY (2018), which established Health and Wellness Centres (HWCs) (now rebranded as Ayushman Arogya Mandirs) to expand screening and early management of CVDs within primary care [38]. CVD reduction targets were further operationalised through the National Multisectoral Action Plan for Prevention and Control of Common NCDs (2017–2022), which adopted a risk-factor reduction approach and emphasised integration within urban health policies [39]. Implementation has relied on converged federal–state leadership, alignment with Ayushman Bharat–PMJAY reforms, the Ayushman Bharat Digital Mission [40], and referral linkages to tertiary care supported by national health insurance. Reputational incentives have also encouraged collaboration with global partners such as Resolve to Save Lives to support IHCI scale-up and policy “best buys”. Policy efforts have primarily focused on strengthening publicly run PHC infrastructure and community entry points. The lesser engagement of private sector structurally weakens such reforms as private sector delivers over 70% of CVD care in India, and drives significant out of pocket expenditures [41, 42]. While updated clinical guidelines have been issued to regulate private providers, enforcement mechanisms remain underdeveloped. Existing public–private initiatives in urban health, including mohalla clinics and tuberculosis control programmes engaging private general practitioners, present potential platforms for extending CVD integration into the private PHC sector.

#### Nepal

Policy concern over the rising CVD and broader NCD burden and its socio-economic consequences was first articulated in the National Multisectoral Action Plan for NCDs (2014–2020) [43]. This plan emphasised cross-sectoral governance, a risk-factor reduction approach, and the positioning of people-centred PHC as the foundation of CVD control. Shortly thereafter, the Ministry of Health and Population endorsed the Nepal NCD and Injuries Poverty Commission to analyse and recommend how NCDs and injuries could be better addressed within national reform processes [44]. In 2016, service delivery reforms were operationalised through the Integrated NCD Care Model, targeting rural PHC facilities. This model implemented the WHO PEN package, including management of hypertension and diabetes, combining community- and facility-based care with strengthened coordination and digital reporting systems.

Renewed focus on hypertension detection and continuity of care has emerged through the Hypertension Care Cascade Programme, piloted in 2023 [45]. More recently, the National Multisectoral Action Plan (2021–2025) [46] and the forthcoming health policy (2026–2035) signal heightened political commitment to CVD control. National implementation is led by the Department of Health Services, which supports the Ministry of Health and Population in developing laws, policies, and strategies for NCD prevention and management. Policy emphasis remains concentrated on strengthening the public PHC network, while strategies to engage the private sector are less clearly articulated.

#### Pakistan

Pakistan was among the first South Asian countries to launch a National NCD Action Plan (2003), followed by a National Action Plan for CVDs (2010–2020). However, implementation stalled due to constitutional devolution of health responsibilities to the provinces [47]. Growing global NCD advocacy and rising domestic concern about the NCD burden and associated costs led Pakistan to endorse UN-supported global NCD targets in 2013 [48]. This was followed by a post-devolution National Framework for NCD Integration into PHC (2015–2020) and the development of provincial NCD action plans. Policy momentum elevated CVDs and other NCDs alongside communicable diseases in the first post-devolution National Health Vision 2016–2025 [49]. Under this framework, a STEPS survey was conducted, CVD clinical protocols were developed [50], and pilot programmes for hypertension control were introduced. UHC reforms further supported development of an Essential Health Services Package, in partnership with the Disease Control Priorities initiative and WHO. The package included community-based CVD risk screening, opportunistic hypertension monitoring, revised essential medicines lists, and expanded treatment for related conditions [51].

Implementation has been constrained by fiscal limitations affecting Essential Health Services Package adoption and by limited PHC restructuring to support early CVD detection and continuity of care. NCD governance combines federal technical stewardship through the national NCD unit with operational design and financing by provincial health departments. Although several provinces have contracted out public PHC facilities to private providers [52], these arrangements have not systematically integrated CVD services or established referral pathways with empanelled hospitals under national health insurance schemes, resulting in missed integration opportunities. More recently, updated policy guidance for CVD and risk-factor management at the PHC level has been provided through the National Action Framework for NCDs and Mental Health (2021–2030) [53] and the forthcoming national health and population policy.

#### Sri Lanka

Sri Lanka was the first South Asian country to reorient its PHC system in response to the epidemiological transition towards NCDs, driven by concern over earlier onset and higher disability from NCDs compared to global averages. CVD integration was led by domestically driven reforms aimed at revitalising government PHC infrastructure, addressing underutilisation, and reducing reliance on private providers for chronic NCD care [54]. The National Policy and Strategic Framework for the Prevention and Control of NCDs (2010) established the foundation for CVD-focused primary care guidelines, revisions to the essential medicines list, screening programmes, and efforts to introduce personal health records [55]. A comprehensive screening programme was introduced targeting adults aged over 35 years, as well as 24–25-year-olds with high-risk histories.

A major structural shift occurred under the 2018 UHC policy, which restructured PHC through the introduction of the Shared Care Cluster Model. This model established apex primary care centres within hospitals to provide frontline CVD services and support satellite facilities for screening and referrals [54, 56]. CVD services were subsequently incorporated into the Essential Health Service Package (2019) [57], although implementation has been constrained by resource limitations. For the first time, the UHC policy also outlined partnerships with private diagnostic centres and pharmacies to address service gaps, alongside contracting-in private general practitioners to extend service hours. However, the extent of implementation remains unclear [58]. The forthcoming health policy (2026–2035) builds on the shared care cluster model, with an expanded focus on life-course CVD risk reduction through a well-being approach.

### Which initiatives have been implemented to integrate CVD services within PHC and what are their characteristics?

Despite clear policy commitments to integrating CVD services within PHC across South Asia, implementation has been uneven. Table 2 summarises key recent initiatives across the region, including both nationally implemented government programmes and smaller pilot or system-level interventions that have not yet been scaled up and are largely funded and implemented by entities external to the region. Initiatives are analysed according to the health conditions targeted, the WHO-defined CVD service functions delivered, the lenses and levels of integration, and the categories of integration applied (Fig. 1).

**Table 2:**
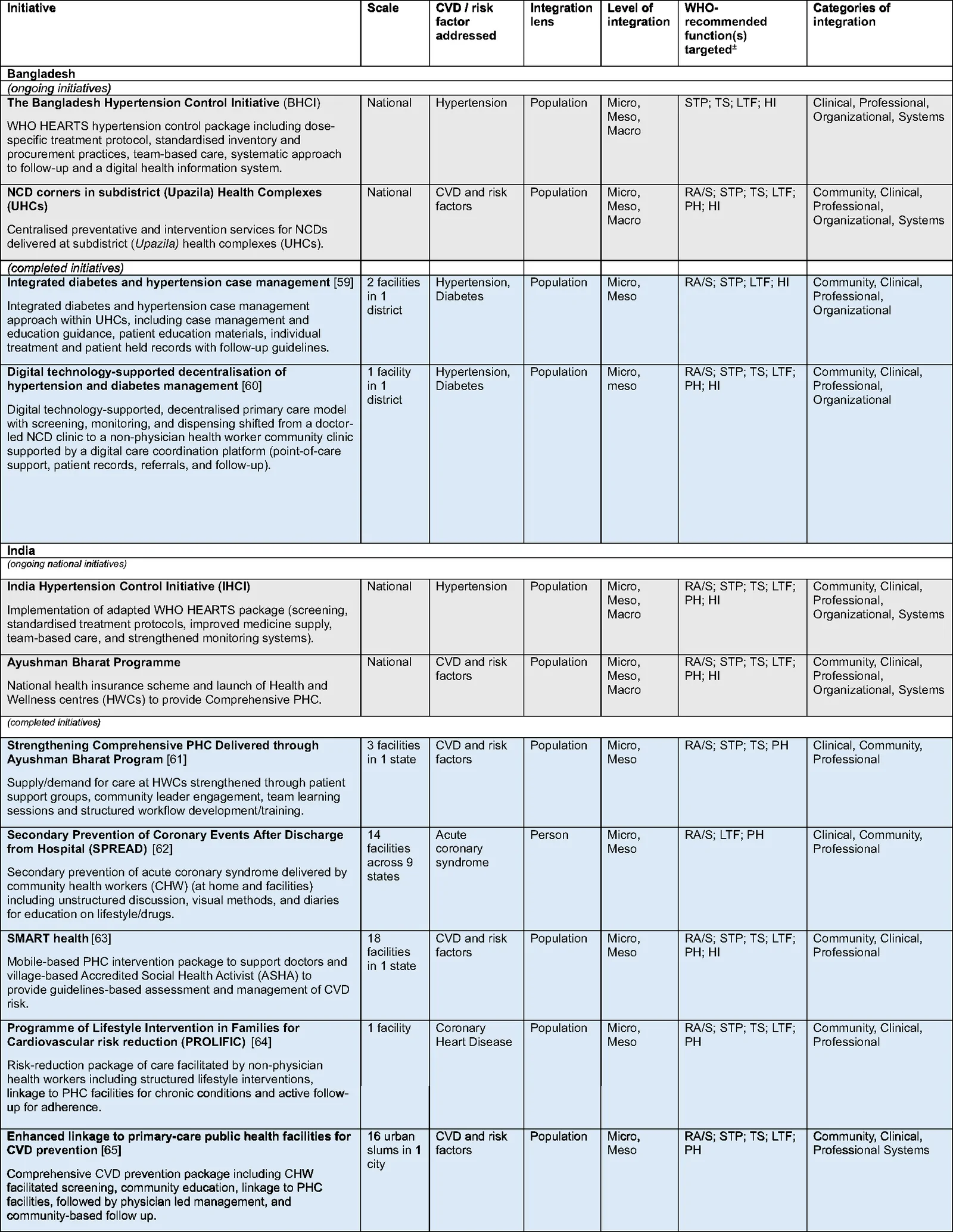

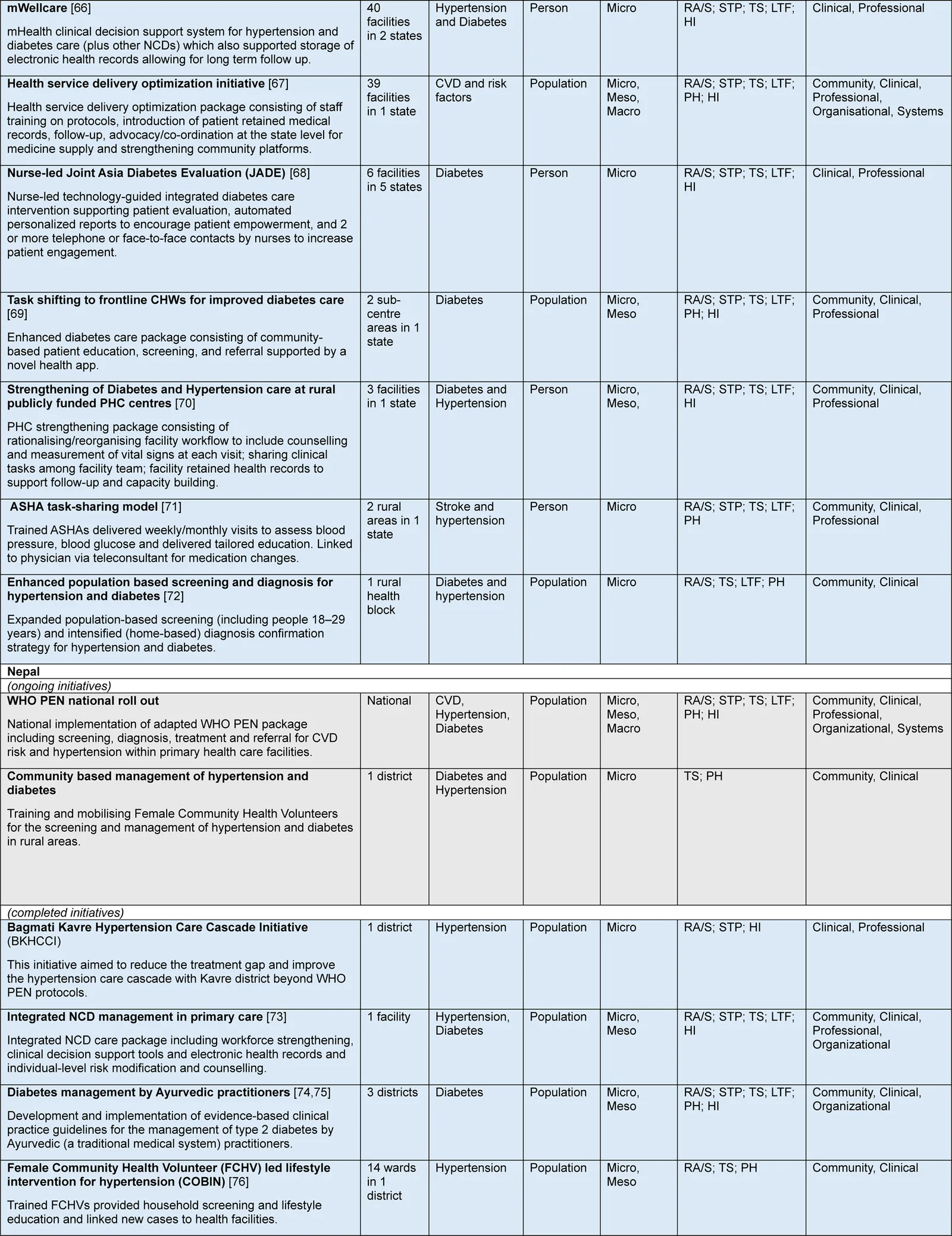

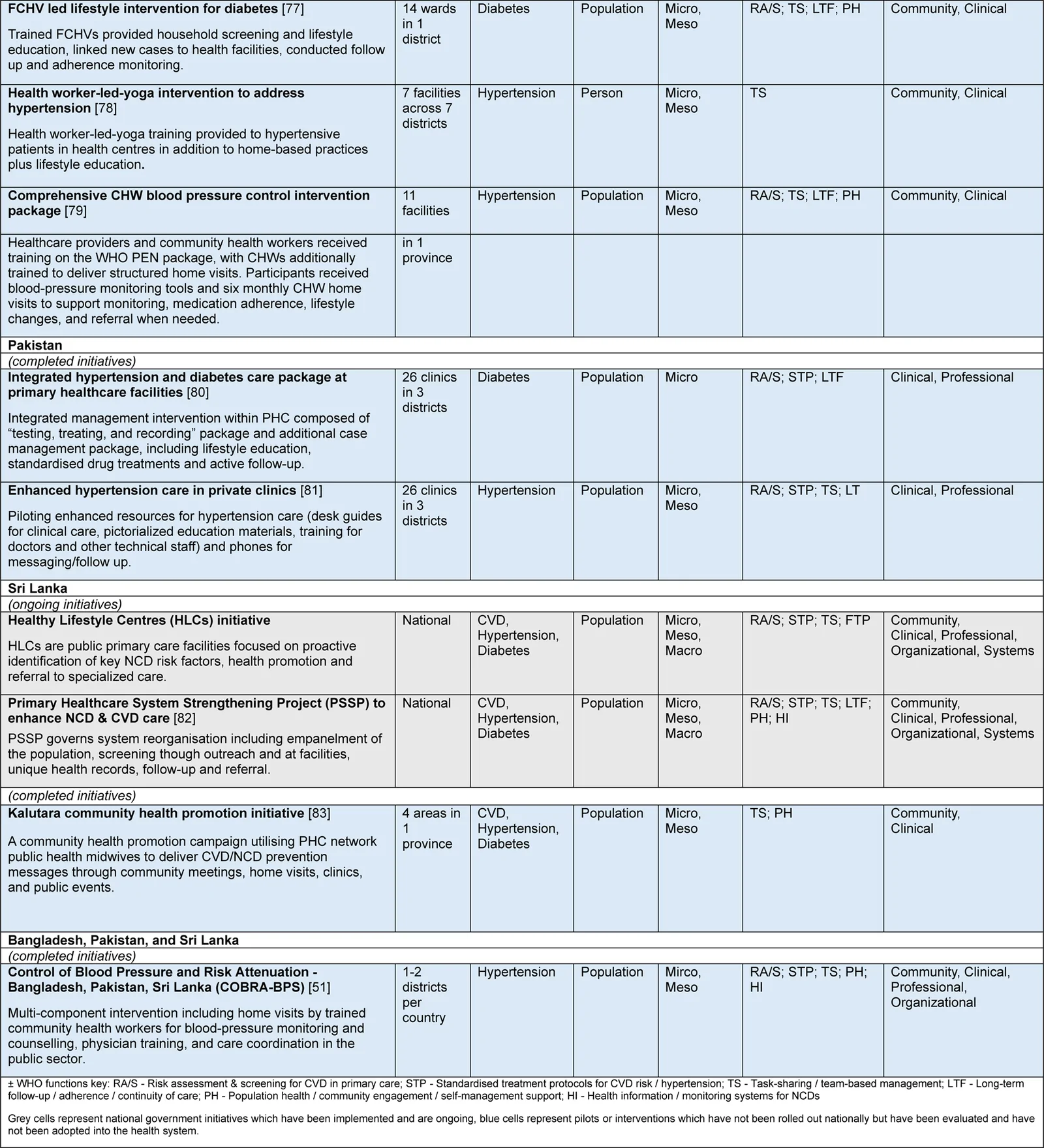
Key recent initiatives integrating CVD services into PHC in South Asia

Several nationally implemented initiatives focus on broadly integrating the prevention and management of CVD and other high-burden NCDs within PHC systems. These include the Health and Wellness Centre reforms (Ayushman Arogya Mandirs) under India’s Ayushman Bharat programme, NCD corners established within Upazila Health Complexes in Bangladesh, Healthy Lifestyle Centres in Sri Lanka, and the national roll-out of the WHO PEN package to the health-post level in Nepal. Complementary national disease-specific initiatives have also been implemented at scale, including the India Hypertension Control Initiative (IHCI) and the Bangladesh Hypertension Control Initiative (BHCI), both launched in 2018. Alongside these national programmes, numerous smaller initiatives and interventions have been developed and piloted across the region (Table 2). Many of these initiatives are also disease specific and focus largely on services for patients with diabetes or hypertension, however in India several pilot programmes have explored integrating follow-up care for patients after acute coronary syndrome [62], stroke [71], or coronary heart disease diagnosis [64] within PHC systems.

Both national programmes and smaller interventions commonly strengthen multiple WHO-defined CVD service functions within PHC. Comprehensive PHC implemented under the Ayushman Bharat programme in India, for example, has expanded guideline-based service delivery to include NCDs. The reforms have also strengthened the workforce through the introduction of Community Health Officers embedded within existing clinical teams to support continuity of care. In addition, community-level assemblies have been used to address social and environmental determinants of ill-health, while information systems have been revamped to support electronic health records [84]. Similar multi-functional integration is evident in smaller programmes. In Bangladesh, a digitally supported decentralisation initiative shifted hypertension and diabetes screening, monitoring, and medication dispensing to village-level community clinics led by non-physician health workers and supported by electronic clinical decision-making, referral systems, and follow-up platforms [60]. However, strengthening of health information systems and monitoring functions has less consistently been implemented.

Most initiatives adopt population-focused approaches to integration. Population groups are typically defined by geography or community membership, for example programmes targeting urban slum populations, to strengthen linkages between community-based prevention activities and PHC service delivery [65]. Person-centred integration is less common but evident in some interventions. The SPREAD intervention in India, for example, delivered community-based secondary prevention following hospital discharge for acute coronary syndrome, focusing on continuity of care for individuals at high cardiovascular risk [62].

In terms of levels at which integration occurs, integration most frequently occurs at the micro and meso levels. At these levels integration often involves strengthening coordination between providers. In Pakistan, for example, mobile-phone-based communication between PHC physicians and allied health professionals improved hypertension follow-up and continuity of care in private clinics [80]. Meso-level integration is often achieved through organisational or workforce reforms. In Nepal, integrated NCD management programmes strengthened coordination between community health workers and PHC providers while introducing digital clinical decision support tools and electronic health records [82]. Macro-level integration is less common but more evident within national reform programmes. In Sri Lanka, the World Bank-supported PHC System Strengthening Project integrated NCD screening and management into primary medical care institutions through improved referral pathways, stronger linkages with Healthy Lifestyle Centres, and upgraded health information systems [82].

Considering the categorisation of integration approaches, community-centred integration is particularly prominent within many initiatives. Programmes frequently leverage established community health worker cadres within national PHC systems, including India’s Accredited Social Health Activists, Female Community Health Volunteers in Nepal, and non-physician community health workers in Bangladesh [60, 71, 76, 77]. Clinical and professional integration is also widely used. The mWellcare intervention, implemented in 40 facilities in northern and southern India, illustrates this approach through an electronic decision-support system that facilitates collaborative care between nurses and physicians, enabling shared review of clinical and lifestyle advice and the generation of individualised care plans [66].

### What are the Critical Barriers to and Enablers of Integrating CVD Services within PHC?

The five South Asian countries are at different trajectories of implementation but face largely similar barriers and enablers which are summarised in Table 3.

**Table 3:** Critical enablers and barriers for integrating CVD services into PHC in South Asia

Physical access	Financial access	Medicine access	Patient information / communication	Home monitoring and technology	Risk factor support
**Bangladesh**					
Enablers:					
Higher readiness at Upazila health complex [85], HEARTS pilots	Free care at public sector tertiary hospitals [86]	Availability of low-cost generics contributed to success of HEARTs pilots [87]	Successful team-based task sharing demonstrated in HEARTS pilots [87]	Pilots with CHWs for home-based monitoring [88]	Digital pilots for risk factor screening and telemedicine support for low-cost access [88, 89]
Barriers:					
Referral concentrated in major cities, non-functional referral pathways; private sector clinics popular but unregulated; parallel unintegrated NGO programmes; interrupted use of informal providers [86, 90]	High OOP expenditure and sparse public budget for downstream NCD services [87] variability in medicine / diagnostic pricing [87]; patient reluctance to pay for diagnostics in pilots [88]	Very limited availability of essential CVD medicines in PHC outlets at large; lack of frontline diagnostics in public sector PHC facilities [85]	Shortages of trained staff in PHC facilities; uneven standard of delivery, fragmented monitoring information systems [85, 86]	Day time CHW visits do not reach male population; hard to track outcomes once out of primary care system [88]	Low community awareness especially in socially deprived groups [91]; weak referral compliance of screened high-risk cases [88]
**India**					
Enablers:					
Equipping of public sector PHC facilities for hypertension control under IHCI; upgraded Family Health Centres for diabetes-hypertension-risk factors under Aardam mission [41, 42]	Free CVD relevant services at PHC; tertiary services at public and private hospitals supported by national health insurance [92]	Availability of protocol drugs for 30 patient days & validated digital BP monitors in IHCI facilities; positive patient experience with both diabetes and hypertension medications at FHCs with [42, 93]	Digitisation reduced paperwork and allowed real-time monitoring in IHCI; Additional staff coordinator managed workflows and records; team cohesion; web-based appointments in FHCs [42, 93, 94]	Medicine reminder systems; Home BP measurement devices; use of polypills; family support for older patients [95]	Various risk screening pilots combing digital tools with facility referrals; weight reduction pilots based on family approach [96, 97]
Barriers					
Distance to health centres in rural locations or long queues in urban areas; uneven population awareness of available services; lack of care pathways for conditions beyond diabetes-hypertension; private provider preference but private clinics not integrated [41, 42]	Travel costs, patients switch between public facilities and private pharmacies [98, 99] Private sector delivers most primary CVD care, but PHC reforms focus on public services with limited private-sector integration. [100]	Logistical issues for prescription refills of >30 patient days ; slow procurement systems under IHCI; doctor reluctance to follow protocol [42]	Staff transfers; time-consuming card and data retrieval in IHCI, lack of national registry [42, 93, 94]	ASHA workers not effectively involved, time constraints for counselling, weak Patient awareness; uneven family support, Patient migration [95, 101]	Weaker adherence from younger patient’s patient difficulty in sustaining behavioural changes; training practitioners in delivering lifestyle interventions; ensuring standardisation in risk screening; reliance on extensive community-based support [93–95]
**Nepal**					
Enablers:					
First-line facilities re-equipped for diabetes, hypertension [102]	Frontline CVD check-ups and medications supported by national health insurance [102]	Availability of essential medicines and glucometers [102, 103]	Provider motivation, training, familiarity with PEN protocol, positive provider-patient relationship [103, 104]	Family member support for adherence [105]	Improved patient perception of CVD risk [105]
Barriers:					
Referral services concentrated in urban centres; public sector PHC facilities do not extended beyond diabetes, hypertension care; private sector clinics unintegrated [102, 106]	Resource constraints for additional CVD relevant services such as rheumatic heart disease screening [103, 106]	Use of traditional/alternative medicines alongside allopathic treatment	Lower health worker awareness in remoter posts; lack of access to clinical updates; staff turnover [103]	Patient awareness of follow-up care 84, 87]; Potential for mobile technology not tapped [105]	Disease and treatment stigma/ fear of disclosure, entrenched lifestyle habits [105]
**Pakistan**					
Enablers:					
Hypertension control and risk screening pilots [51]	Free CVD tertiary care at public & private hospitals through national health insurance, special CVD centric hospital initiatives	On site glucose testing & first line CVD relevant essential medicines in PHC system [107]; PHC pilots involving appropriate prescribing and annual retraining of physicians contribute to successful outcomes [51]	Pilots for hospital-based registries, structured clinical assessments with ACVD scoring [108]	Family support for adherence; Digital pilots drawing on high smartphone penetration and WhatsApp use [109]	Pilots involving longer duration CHW follow up with back-up referral to equipped PHC facilities and CHW annual refresher training [51]
Barriers:					
Sparse CVD services outside urban tertiary hospitals; emergency ambulances in urban centres but travel barriers for rural referrals; lack of referral pathways; private clinics often first point of care but unintegrated [110, 111]	CVD funding concentrated at tertiary hospitals; out of pocket expense leads to interrupted primary care for CVDs [111]	Lack of ACE inhibitors in EDL; Highly variable supply of essential medicines across districts; absence of basic CVD diagnostic support in PHC system [107]	Fragmented information systems, lack of national registries; physician reluctance to refer; population migration affects follow-up [110, 112]	Uneven digital literacy; variation in adherence across social groups; CHW workload [109]	
**Sri Lanka**					
Enablers:					
Shared cluster models, pilots involving specialized downstream diabetes centres [54, 113] Strong policy commitment for primary care reforms with establishment of Community Arogya centres Community health workers visiting home, engaged in family education and prompting health seeking behaviour	Free care at public sector tertiary hospitals, downstream diabetes clinics	Access to medications through public sector or affordable private pharmacies; standardized ADA therapeutic protocols [54, 113]	Physician’s ability to communicate well, physician retraining [54, 113]	Family support; Patient awareness of hypertension as a serious condition motivating care-seeking; availability of community health workers providing education and outreach [114, 115]	Piloting of machine-based predictive risk stratification tool [103]
Barriers:					
Referrals services concentrated in urban centres, emergency ambulances in urban centres but travel barriers for rural referrals; lack of referral pathways; private clinics often first point of care but unintegrated [54, 116, 117]	High out-of-pocket expenses on private care, travel leading to interrupted CVD treatment [114]	Medicine shortages; Use of traditional medicines alongside allopathic treatment [54, 117]	Deficient health information and referral tracking; Variation in counselling/ communication practices Digitalized system, is envisaged to support better patient care, continuity of care, follow up etc., this has been a relatively slow contributor to the overall reform process	Potential issues related to health literacy and adherence [114]	Poor control of cardiovascular risk factors; Low patient knowledge of CVD and asymptomatic nature ensuring standardisation in risk screening; reliance on extensive community-based support [113, 114, 118]

#### Physical and Financial Access

Access to upgraded PHC centres varies by location, as more rural areas face travel constraints, whereas other areas at times face overcrowding, requiring future efforts to support more downstream care. Care coordination is the biggest challenge regionally, as private clinics and pharmacies remain popular but are not sufficiently integrated within the larger system; CVD referral centres are in major urban centres, creating access issues, and referral pathways are absent [41, 42, 54, 86, 90, 102, 106, 110, 111, 116]. Financial access to frontline CVD care and medications has been made available through national health insurance in Nepal and PHC reform initiatives in India, however there are resource constraints for additional CVD-related services [92, 102]. There has been little fiscal reform to public sector expenditure in Pakistan, Sri Lanka, Bangladesh for downstream CVD control and prevention services. Resourcing remains concentrated in public sector tertiary hospitals with significant OOP expenditure on accessing CVD-related care including travel, diagnostics and medicines leading to interrupted care [87, 88, 111, 114].

#### Medicine Access

Medicine access is a critical enabler of patient adherence however medicines have been traditionally constrained by supply shortages in public-sector PHC facilities [54, 85, 107, 113] and essential drug lists (EDLs) have not been adapted to include full protocol drugs such as ACE inhibitors [107]. Evidence shows that the availability of low-cost full protocol generics in HEARTS pilots in Bangladesh and the provision of additional medicines beyond those on the EDL, accompanied by annual physician retraining in hypertension control pilots in Pakistan, Sri Lanka, and Bangladesh, served as critical enablers for effective delivery of CVD services within PHC [51, 54, 87, 113]. However, the pilots also showed that patients were reluctant to pay for diagnostics. Experience from upscaled programmes in India and Nepal underscores the ability to provide patients with a minimum of one month supply of all protocol drugs, onsite glucometers and validated digital BP monitors, although logistical issues constrained prescription refills beyond 30 days, as well as slow procurement and instances of doctor reluctance to follow protocol for prescribing additional medications [42, 93, 102, 103].

#### Patient Information and Communication

Follow-up information and communication experience shows contextual variations in delivery but key commonalities in terms of critical enablers. Successful team-based cohesion is evidenced in India’s upscaled programme delivered through Family Health Centres [42, 93, 94] and HEARTS pilots in Bangladesh [87], provider motivation and positive patient-provider relationships from PEN package implementation in Nepal [103, 104], quality of communication by physician in multi country hypertension pilots, as well provider familiarity with protocols in initiatives across all five countries. Creation of additional staff posts of facility-based care coordinators have helped with workflows, reporting and follow-up in FHCs in India as well as various country pilots. Staff transfers, uneven counselling practices and weak training in remote posts emerge as key constraints highlighting necessity for continuous re-training with a focus on patient messages (Table 3). All five countries have traditionally lacked information systems for CVD and NCD tracking. Digitisation of records, as seen in the IHCI initiative in India, has helped in allowing real time tracking of cases and follow-ups, whereas there have been confined attempts to create hospital-based registries in Pakistan [42, 93, 94, 110, 112]. However fragmented information systems across PHC and referral tiers, and lack of national registries constrain follow up of patient outcomes especially once they leave the primary care setting as seen implementation of large-scale initiatives as well as pilots [42, 93, 94, 110, 112]. 

#### Home-Based Monitoring

Home-based monitoring attempts have been enabled with CHWs, medicine reminder systems and digital technology in Bangladesh, India, Nepal, Pakistan and Sri Lanka [88, 95, 109, 114, 115]. Community-clinic linkage remains a challenge across smaller pilots as well as large upscaled initiatives. CHWs effectiveness in home-based support is constrained by role definition, workload issues, training and time constraints for counselling as reported from India’s IHCI and smaller pilots in Pakistan [95, 101, 109], whereas Bangladesh experience shows that CHW’s daytime visits have not been effective in reaching the male population [88]. This requires fresh thinking on new modalities of reaching patients and communities. Digital home monitoring pilots have been implemented in different settings drawing on high WhatsApp use and smart phone penetration as key enablers particularly in India and Pakistan despite variation in digital literacy. Evidence on usability and feasibility factors is not available but a key area for future research. Weak patient awareness is a common critical constraint across all five countries for follow-up adherence and home-based care and indications of uneven adherence across social groups (Table 3). Family support for elderly patients where seen has enabled follow-ups.

#### Risk Factor Screening

Risk factor screening attempts have been piloted involving machine learning based digital risk stratification tools [103], whereas other have deployed a family-based approach [96, 97]. Studies report that provision of back-up referral access to PHC facilities or telemedicine facilities can be important enablers [51], however there are reported concerns on referral compliance of screened cases and reliance on extensive community-based support that may not be feasible to replicate at scale [93–95]. Entrenched lifestyles, denial of risk are commonly reported whereas at least one study reported that younger population experience more difficulty in post risk follow up and sustaining behavioural changes [93–95]. Training of practitioners on delivering lifestyle interventions has been suggested from reported studies but larger multi-sector action for risk factor awareness and counter measures are required with context specific strategies.

### What are the impacts of integrated CVD services on health and health system outcomes?

Finally, we extracted recent evidence exploring what impact the integration of CVD services within PHC has had on key health and health service outcomes in the region. Characteristics of several key studies from selected South Asian countries are highlighted in Table 4.

**Table 4:**
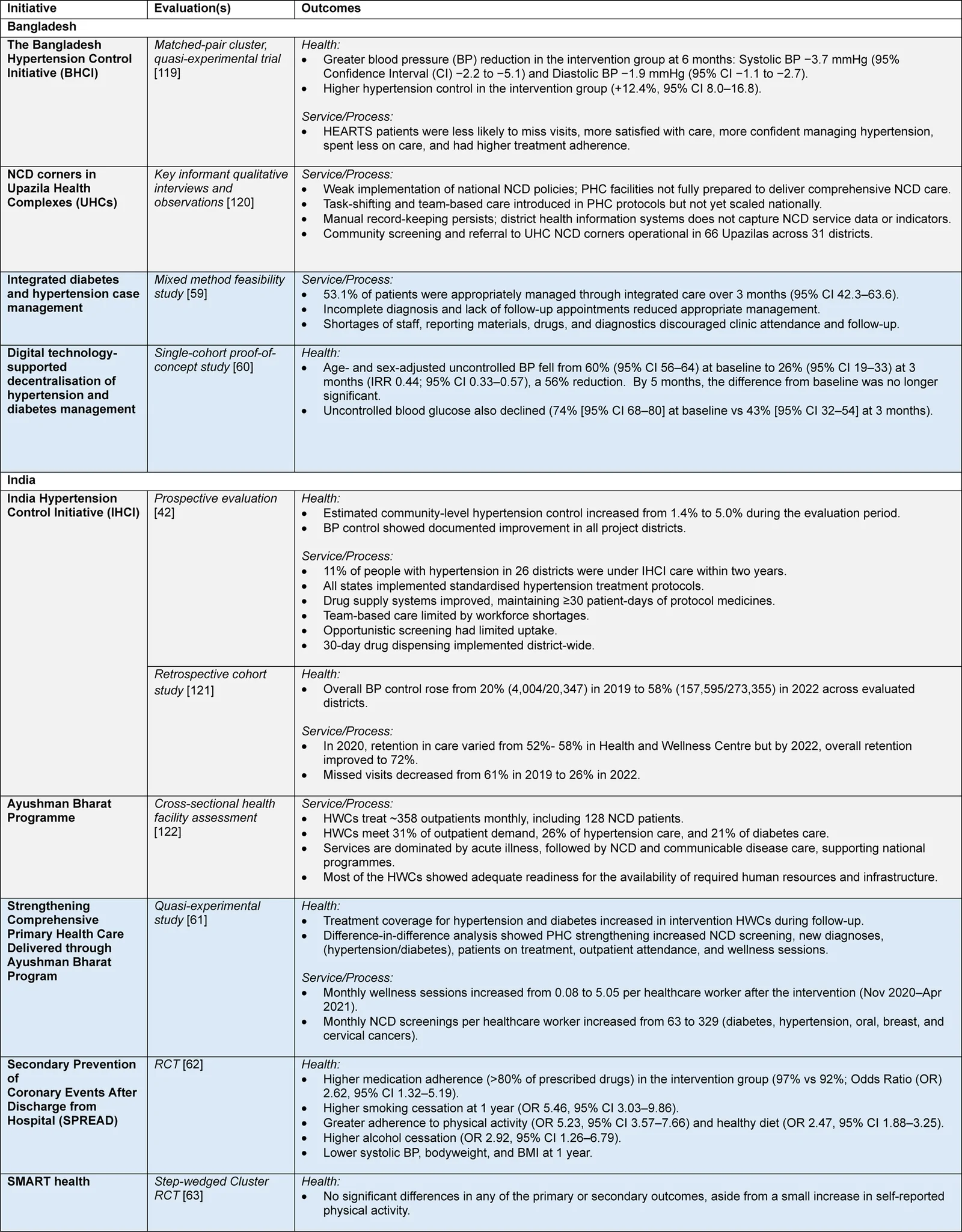

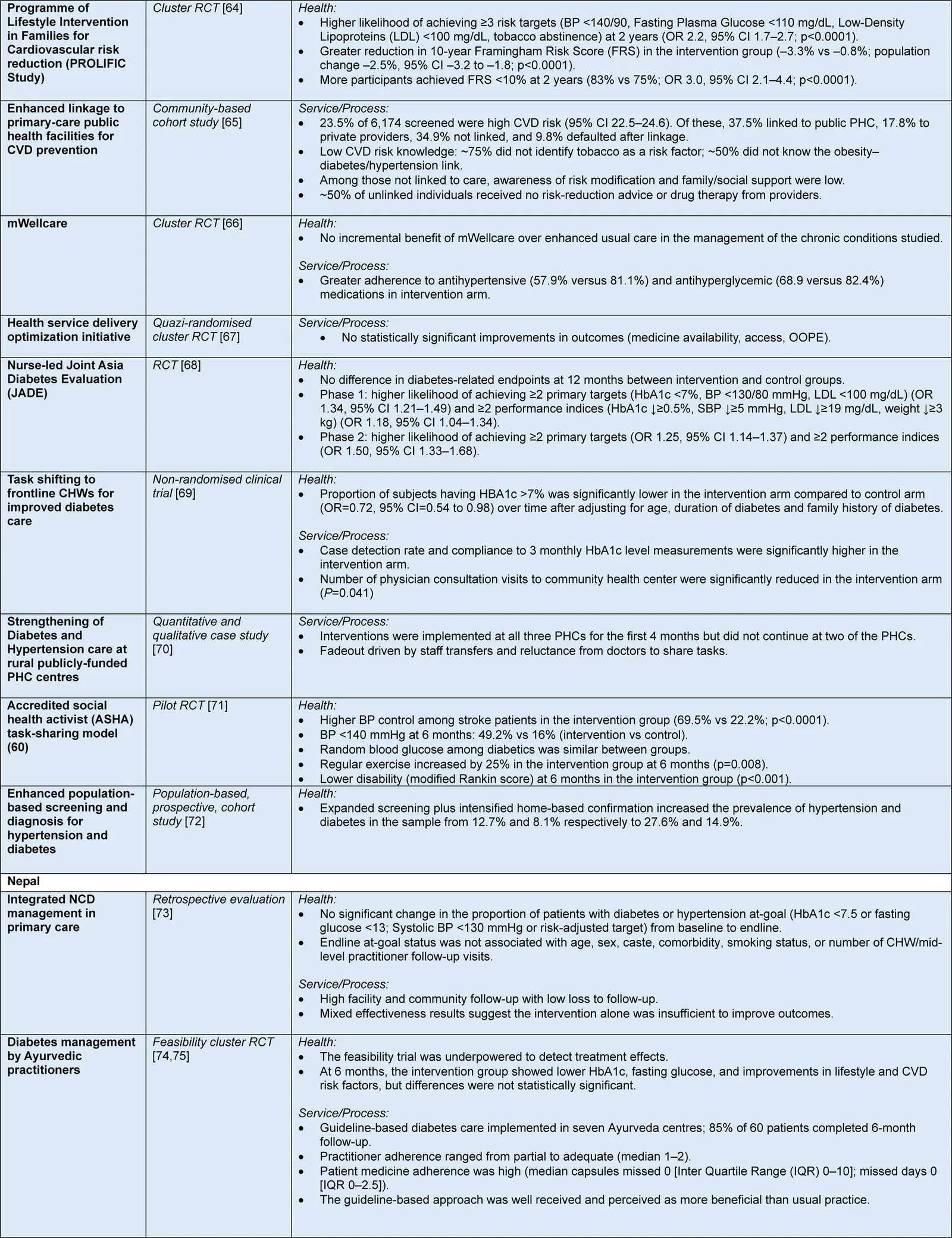

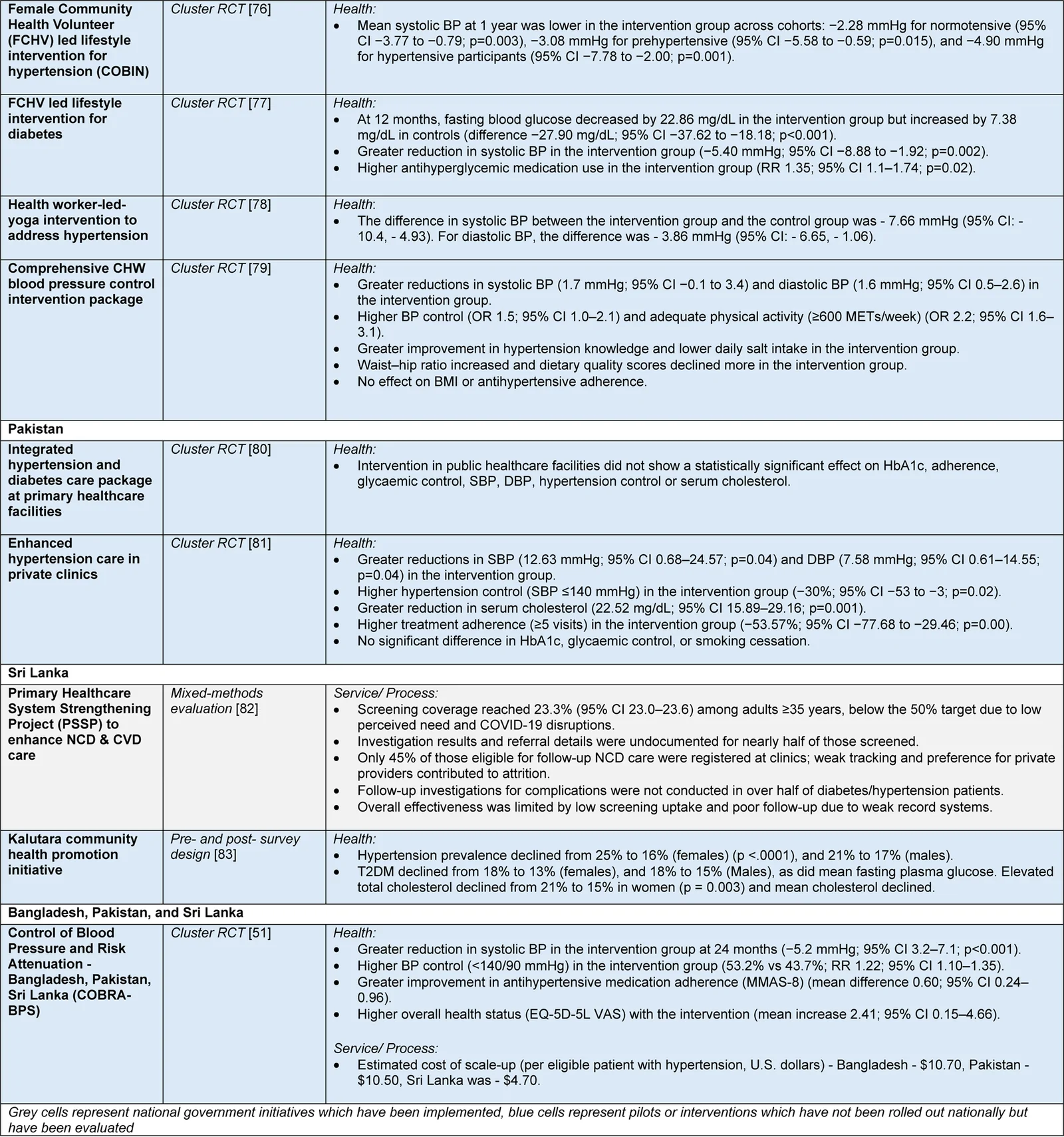
Evidence of the impact of the integrating CVD services in PHC on health and process outcomes within South Asia

Contemporary evidence for the impact integrated CVD care has largely been generated in India, whilst more limited research has been conducted in Pakistan, Bangladesh, Nepal and Sri Lanka. Importantly, both the methods utilised and integrated CVD care approaches evaluated within recent studies are highly heterogenous (Table 4). Comprehensive evaluation of integrated CVD care at the national level is largely absent, with some notable exceptions. Most contemporary evidence has explored the impact of pilot or novel approaches to integration at the district or subdistrict level, prior to implementation within the wider health system.

#### Health Outcomes

Given the heterogeneity of settings, study designs and approaches to integrated CVD care evaluated across the region, summary of impacts on health and health service outcomes should be interpreted cautiously. Considering national level initiatives, both prospective and retrospective evaluations of the IHCI showed positive impacts on health [42, 121]. Kaur et al. [42] evaluate the implementation if IHCI across 26 districts in five states finding that average community-level hypertension control increased from 1.4% to 5.0% between January-March 2019 and January-March 2020, with 13 districts exceeding 5%, whilst Chavan et al. [121], who evaluated hypertension treatment records in in over 4,000 public facilities from nine districts in the two states, reported blood pressure control rose from 20% in 2019 to 58% in 2022. In Bangladesh, a matched-pair cluster, quasi-experimental trial conducted in UHCs within two rural districts, found that, compared to usual care, WHO HEARTS package implementation under BHCI expansion led to a greater reduction in both systolic and diastolic blood pressure in addition to greater hypertension control (Table 4) [119].

For smaller scale initiatives, several randomised and non-randomised experimental studies reported a significant positive impact of integrated care interventions on patient health outcomes [51, 61, 62, 64, 68, 69, 71, 76–79, 81]. Predominantly these were conducted in India and Nepal, in addition to one study in Pakistan and one multi-country (Bangladesh, Pakistan, and Sri Lanka). Largely, evaluated outcomes can be considered as intermediate clinical risk factors for CVD onset and include systolic blood pressure, blood glucose and low-density lipoprotein (LDL). The PROLIFIC trial, conducted in India, evaluated a family-focused cardiovascular risk reduction intervention in individuals with a family history of premature coronary heart disease that led over a two-fold odds increase in a composite primary outcome representing reduction in cardiovascular risk and risk behaviours [64]. Similarly, the JADE trial, also conducted in Inda, which evaluated a technology-guided nurse-led structured evaluation tool in addition to patient empowerment messages and additional follow-up, found a reduction in cardiometabolic risk factors for CVD including reduction of LDL cholesterol and HbA1c [68]. However, the intervention had no significant impact on endpoints associated with diabetes, including cardiovascular disease and death [68]. We did not identify any other studies evaluating the impact of integration on mortality or disease incidence. Conversely three large scale trials reported no significant impact on evaluated health outcomes [63, 66, 80].

#### Service and Process Outcomes

As with health outcomes, due to the heterogeneity of both initiatives and evaluation designs there is substantial variation in process or service level outcomes evaluated (Table 4). Most commonly, outcomes related to screening coverage or case-detection rate, retention in care and follow-up, treatment adherence/knowledge and system linkages.

Two initiatives conducted in India, were found to meaningfully increase rates of screening activities [61] or case-detection [69] for drivers of cardiovascular disease. Notably, interventions to strengthen Comprehensive Primary Health Care delivered through Ayushman Bharat program were found to increase the number of monthly screenings delivered at HWCs by over 250 visits (63.04 (95% CI: 20.36–105.73) at baseline vs. 328.56 (95% CI: 125.36–531.75) in the follow-up period) [61]. However, a similar initiative in Sri Lankan titled the Primary Healthcare System Strengthening Project (PSSP) failed to achieve targeted screening coverage following implementation due to due to lack of perceived need for screening among the public and COVID-19-related service disruptions [82].

Studies evaluating initiatives in India, Sri Lanka, Bangladesh and Nepal appraised the impact of integrated care on retention and/or follow-up [59, 73, 82, 119, 121]. Implementation of the IHCI led to a 33% point reduction in missed visits across two states in India [121] whilst patients receiving the HEARTS intervention through BHCI were significantly less associated with likely to miss scheduled clinic visits (IRR 0.22, 95% CI 0.15 to 0.32), and more likely to have a clinic visit < 3 months after enrolment (IRR 1.67, 95% CI 1.49 to 1.87) [119]. An integrated NCD management in primary care initiative in Nepal experienced relatively low loss-to-follow up within hypertensive and diabetic patients (19% and 16% over 18 months) suggesting successful implementation [73]. Conversely, the Primary Healthcare System Strengthening Project to enhance NCD and CVD care initiative in Sri Lanka demonstrated very high loss-to-follow up due to significant issues with documentation and referral processes [82] whilst low levels of follow-up for Integrated diabetes and hypertension case management in Bangladesh was attributable to poor clinical management and human and clinical resource shortages [59].

Alongside improvements in retention, patients receiving care through the BHCI were also more adherent to antihypertensives to those in usual care (95.4% in the intervention group vs. 75.7%, *p* < 0.001) [119] and despite not leading to impacts on measured health outcomes, mWellcare in India led to a significant increase in self-reported adherence to both antihypertensive (57.9% versus 81.1%) and antihyperglycemic (68.9 versus 82.4%) medication [66].

Finally, whilst small scale initiatives centring Community Health Workers (CHWs) within integrated preventative or curative treatment delivery supported linkages with primary healthcare centres [65] and reduced unnecessary physician visits [69], others were impacted by existing system weaknesses. Within NCD corners of Bangladesh’s UHCs key informants describe poor implementation of national NCD policy due to insufficient information infrastructure, poorly functioning referral systems and human resource constraints [120]. Similar systems challenges, particularly related to drug procurement, within implementation of IHCI threatened to impact implementation however initiative processes around forecasting, budget allocation and distribution ensured one-month refills continuously for each Patient for protocol drugs during evaluation [42].

## Discussion

This review has evaluated progress towards the integration of CVD services within PHC systems in five of South Asia’s most populous countries through synthesis of relevant policy documentation and empirical research. Findings from this review highlight the substantial public policy attention towards the integration of both CVD and broader NCD services within existing PHC systems across South Asia in recent years. This focus is driven largely by shifting epidemiolocal profiles, as the proportion of disease burden attributable NCDs continues to increase, triggering growing national concern and further strain on existing health systems. However, meaningful engagement with the private sector is often absent within this policy space, despite its dominance within curative primary care in South Asian nations [21, 22]. Looking forward, governments must continue to engage with the private sector to ensure CVD services are effectively integrated into PHC including by aligning private sector partnerships with national PHC policies [23]. This will require significant centralised oversight and governance structures which have previously been found to be lacking across key countries in the region [20]. Furthermore, progress towards integration must accommodate traditional and indigenous medicine systems [23] which are both hugely prevalent across the region and closely embedded into community healthcare systems. Whilst key evidence is emerging in recent years [74, 75, 78], further culturally sensitive empirical evaluation of how these systems and practices can support progress toward integrated CVD care is vital.

Despite this wide-spread explicit policy commitment, implementation of integrated CVD services within PHC is highly varied, with countries such as Pakistan experiencing limited national scale up of integrated CVD care, whilst others have developed national programmes both within existing programmes as well as distinctive CVD risk-factor control initiatives. These findings mirror evaluations of global progress towards implementing the WHO HEARTS packages, which, whilst highlighting substantial progress in improving cardiovascular health across LMICs, demonstrate significant variability in successful implementation across countries due to health system constraints [123]. Nevertheless, we emphasise that in countries such as India and Bangladesh, longstanding and nationally scaled up initiatives not only exist, but also seek to ensure that preventative and curative CVD services are positioned at the heart of community-focused PHC systems. Broadly, the characteristics of these initiatives, and of many other smaller scale pilots and interventions across the region, exemplify approaches to integration that seek to strengthen organisational and professional linkages within PHC systems by utilising existing health system capabilities including networks of community or non-physician health workers. Continued utilisation of the region’s diverse community-based health workforce, who can spearhead health promotion and prevention, will be essential to further progress towards PHC-focused integration given the breadth of roles CHWs play within regional systems and the current underutilisation within the delivery of NCD services [124].

Crucially, our findings demonstrate that significant barriers towards integration both exist and share similarities across the region. From a systems perspective, fragmentation within urban PHC systems, and limited public PHC facilities are key barriers to truly integrated CVD care given the regions rapid urbanisation and growing CVD burden. Referral pathways between PHC facilities and secondary or tertiary care constitute a significant weakness within health systems which will require targeted reform to improve poor health outcomes. Beyond identified barriers, recent political turbulence exemplified by regional youth-led protests, contested democratic transitions and border conflicts has the potential to stymie further progress [125]. Nevertheless, evidence suggests that key enablers exist within South Asian health systems to support moves towards integrated CVD care. These include national insurance and PHC reforms which have improved financial access to frontline CVD care and medicines within the sector, evidence of effective and cohesive team-based models of care and enhanced engagement with telemedicine and digital systems.

Finally, our findings also demonstrate that understanding the impact of integrated care approaches on health services and health outcomes in South Asia is complicated by significant heterogeneity in integrated care models and methods of evaluation. Evidently, integrated care can improve intermediate CVD clinical risk factors, such as hypertension, LDL cholesterol and HbA1c, and important service delivery outcomes such as screening coverage, follow-up and treatment adherence. However, evidence on systems level assessment of impact on vital endpoint outcomes such as CVD incidence or mortality is missing. Large scale evaluations of national initiatives are rare with much empirical evidence focused on the evaluation of small-scale system interventions.

## Conclusion

Integrating CVD services within PHC systems is central to national health policy across South Asia as countries respond to rising disease burdens and fiscal constraints. Although implementation varies, several national initiatives, often leveraging community-based service delivery, demonstrate that CVD prevention and management can be effectively delivered through PHC while strengthening system organisation and continuity of care. However, barriers to wider implementation reflect broader health system constraints common across low- and middle-income countries. While empirical studies have evaluated integrated CVD care in the region, further research is needed to assess impacts on CVD morbidity and mortality to inform continued improvements in care.

## Key Studies

- Ahmed SM, Krishnan A, Karim O, Shafique K, Naher N, Srishti SA, et al. Delivering non-communicable disease services through primary health care in selected south Asian countries: are health systems prepared? Lancet Glob Health. 2024;12(10):e1706–19. doi: https://doi.org/10.1016/s2214-109x(24)00118-9.

- ○ Regional evaluation systematically exploring health system readiness to provide NCD though existing PHC systems.

- Kaur P, Kunwar A, Sharma M, Durgad K, Gupta S, India Hypertension Control Initiative collaboration, et al. The India Hypertension Control Initiative-early outcomes in 26 districts across five states of India, 2018–2020. J Hum Hypertens. 2023;37(7):560–7. doi: https://doi.org/10.1038/s41371-022-00742-5.

- ○ First multi-state analysis evaluating the ongoing government-led PHC reform which integrates CVD risk factor control into primary care within India.

- Jafar TH, Gandhi M, Silva HA de, Jehan I, Naheed A, Finkelstein EA, et al. A Community-Based Intervention for Managing Hypertension in Rural South Asia. N Engl J Med. 2020;382(8):717–26. doi: https://doi.org/10.1056/nejmoa1911965.

- ○ Evaluation of a multicomponent PHC intervention which integrated screening and referral into existing system structures in three South Asian countries and led to significant reduction in hypertension.

## Data Availability

No datasets were generated or analysed during the current study.
